# Association of anthropometric indices with the development of multimorbidity in middle-aged and older adults: A retrospective cohort study

**DOI:** 10.1371/journal.pone.0276216

**Published:** 2022-10-14

**Authors:** Shuoji Geng, Xuejiao Chen, Zhan Shi, Kaizhi Bai, Songhe Shi

**Affiliations:** 1 Department of Epidemiology and Health Statistics, College of Public Health, Zhengzhou University, Zhengzhou, Henan, People’s Republic of China; 2 Department of pharmacy, Zhengzhou people’s hospital, Zhengzhou, Henan, People’s Republic of China; Universidade Federal do Rio Grande do Sul, BRAZIL

## Abstract

**Background:**

Previous studies have explored the relationship between body mass index (BMI) and multimorbidity. However, the relationship between other obesity indicators and their dynamic changes and multimorbidity has not been systematically estimated. Therefore, we aimed to investigate the association of BMI and other obesity indicators, including waist circumference (WC), waist-to-height ratio (WHtR), waist divided by height^0.5^ (WHT.5R), and body roundness index (BRI) and their changes and the risk of multimorbidity in middle-aged and older adults through a retrospective cohort study.

**Methods:**

Data collected from annual health examination dataset in the Jinshui during 2017 and 2021. Cox regression models were used to calculate hazard ratios (HRs) and 95% confidence intervals (CIs) to evaluate the effect of baseline and dynamic changes in the anthropometric indices on the risk of multimorbidity.

**Results:**

A total of 75,028 individuals were included in the study, and 5,886 participants developed multimorbidity during the follow-up. Multivariate Cox regression analysis revealed a progressive increase in the risk of multimorbidity with increasing anthropometric indicators (BMI, WC, WHtR, WHT.5R, and BRI) (all *P*<0.001). Regardless of general obesity status at baseline, increased WC was associated with a high risk of multimorbidity. Compared to the subjects with baseline BMI<24 kg/m^2^ and WC<90 (men)/80 (women), the HRs (95% CI) of the baseline BMI<24 kg/m^2^ and WC≥90 (men)/80 (women) group and BMI≥24 kg/m^2^ and WC≥90 (men)/80 (women) group were 1.31 (1.08, 1.61) and 1.82 (1.68, 1.97), respectively. In addition, the dynamics of WC could reflect the risk of multimorbidity. When subjects with baseline WC<90 (men)/80 (women) progressed to WC≥90 (men)/80 (women) during follow-up, the risk of multimorbidity significantly increased (HR = 1.78; 95% CI, 1.64, 1.95), while the risk of multimorbidity tended to decrease when people with abnormal WC at baseline reversed to normal at follow-up (HR = 1.40; 95% CI, 1.26, 1.54) compared to those who still exhibited abnormal WC at follow-up (HR = 2.00; 95% CI, 1.82, 2.18).

**Conclusions:**

Central obesity is an independent and alterable risk factor for the occurrence of multimorbidity in middle-aged and elderly populations. In addition to the clinical measurement of BMI, the measurement of the central obesity index WC may provide additional benefits for the identification of multimorbidity in the Chinese middle-aged and elderly populations.

## Introduction

Multimorbidity (the coexistence of two or more chronic diseases) is an increasing global public health concern [1]. Investigations indicated that a quarter of adults have multimorbidity, with some estimates as high as 98% among older adults [2, 3]. Compared to those with one or no chronic diseases, people with multimorbidity are at an increased risk for functional decline [4] and mortality [5], poorer quality of life [6], and greater health care use [7]. Epidemiological studies have found that multimorbidity is common in middle-aged and elderly people, especially among the obese population [8]. As the largest developing country, China also faces serious challenges due to its large chronic disease burden [9]. Evidence has shown that 43.6% of community-dwelling people over the age of 60 in China have multimorbidity [10]. Therefore, identifying the high-risk population of multimorbidity early is crucial to preventing multimorbidity and improving prognoses.

Obesity is recognized as a major alterable risk factor for the development of multimorbidity [11]. Thus, monitoring changes in obesity is of medical importance to prevent the development of multimorbidity. As anthropometric measurements are a widely used, noninvasive, and cost-saving public health tool, the search for more effective anthropometric indicators associated with the risk of developing multimorbidity has important clinical and public health implications. Currently, BMI is still the most widely used indicator to measure general obesity. In addition, central obesity has been of increasing interest to a wide range of researchers. Numerous studies have shown that central obesity is more closely associated with metabolic syndrome and cardiovascular diseases (CVD) than general obesity and is also an independent risk factor for mortality [12, 13].

Central obesity includes several established and novel indices. Waist circumference (WC) is a commonly used clinical indicator that is strongly associated with the development of CVD, diabetes, hypertension, cancer, and kidney diseases [14, 15]. However, WC does not take into account differences in height and therefore may lead to overestimating the risk for taller people or underestimating the risk for shorter people. The waist-to-height ratio (WHtR) balances the effect of height in addition to WC and is an alternative measurement for visceral fat [16], and is considered a better screening tool for diabetes, hypertension, and CVD than BMI and WC [17]. Waist divided by height^0.5^ (WHT.5R) is a new waist circumference ratio indicator that excludes the effects of height and elements of adiposity and is considered an optimal predictor of cardiovascular disease [18]. In addition, a new geometric index that combines height, waist circumference (WC), and hip circumference (HC) has been proposed and used, specifically, the body roundness index (BRI) [19]. Some studies have revealed that BRI is significantly associated with the occurrence of metabolic syndrome, insulin resistance, and CVD [20, 21]. Although the above measures have been widely used in various studies, most of the previous studies were based on a cross-sectional design and tended to focus on the association between a single indicator of obesity and multimorbidity and the relationship between different obesity indicators and their dynamic changes and multimorbidity has not been systematically estimated.

Therefore, the present study aimed to compare the correlation between baseline and changing trends in BMI, WC, WHtR, WHT.5R, and BRI and the development of multimorbidity in Chinese adults aged 45 to 85 years.

## Methods

### Study design and participants

The present study was based on an annual health screening dataset from the Electronic Health Management Center of Jinshui District, Zhengzhou City, Henan Province, China. The annual health screening program in Jinshui District is organized and led by the Zhengzhou Health Commission and is an important part of China’s basic public health service program. The survey is organized and implemented by the Zhengzhou Community Health Centre. From January 2017 to October 2021, the study cohort included 120,218 subjects with ≥2 physical examination data and no multimorbidity at baseline. From these 120,218 participants, we first excluded 24,560 participants aged <45 years and >85 years. Then, we excluded those with less than one year of follow-up (n = 7,505), those with missing data regarding marriage, height, weight, WC, smoking, drinking, or physical activity at baseline or follow-up (n = 13,088), and those who died during follow-up due to disease or other causes (n = 37). The Inclusion criteria in this study were as follows: (1) with ≥2 physical examination data; (2) with no multimorbidity at baseline; (3) aged 45 to 85 years; (4) follow-up time more than 1 year; (5) no missing data for marriage, height, weight, WC, smoking, drinking, or physical activity. Finally, a total of 75,028 participants were included in the retrospective analysis (S1 Fig). The Ethics Committee of Zhengzhou University approved the administration of this study (Reference Number: ZZUIRB2019-019), and the study team received permission to use the data granted by the Zhengzhou Health Committee.

According to the Working Group on Obesity in China (WGOC), overweight and obesity were defined as 24kg/m^2^≤BMI<28kg/m^2^, and BMI≥28kg/m^2^, respectively [22]. Central obesity was defined as WC≥90 cm in men and WC≥80 cm in women as defined by the International Diabetes Federation [23], and elevated WHtR was defined as ≥0.5 [16]. Due to the lack of uniform classification criteria, 6.76 and 4.84 were chosen as thresholds for WHT.5R, and BRI, respectively, with calculations being based on the Youden index.

Information on common chronic diseases diagnosed by residents or self-reported by subjects was recorded as follows: (1) hypertension, (2) diabetes, (3) heart disease (including myocardial infarction, coronary heart disease, angina, congestive heart failure, or other heart problems), (4) stroke, (5) chronic obstructive pulmonary disease (COPD), (6) cancer (including any malignant tumors except for skin cancer), and (7) psychiatric problems. In the present study, multimorbidity was defined as the presence of two or more chronic diseases in a person, which is consistent with previous studies [24–26].

### Data collection

To collect information, trained medical staff organized by hospitals and community health centers create health records for residents who attend annual physical examinations. Marital status was divided into couple and single, where single included unmarried, divorced, widowed, or others. The study population was divided into nonsmokers and previous/current smokers according to the criteria of having smoked at least 100 cigarettes in their lifetime and whether they were current smokers [27]. Drinking was divided into two categories: current and former/never. Physical activity was classified as yes or no according to whether the frequency of exercise was greater than one time per week. The study subjects wore light clothing and bare feet for height, weight, and waist measurements [28]. Body height and weight were measured via a standard digital weighing scale and stadiometer, respectively. WC was measured using a calibrated tape measure while the subject was standing and during slight expiration.

Based on height, weight, and waist circumference information, BMI, WHtR, WHT.5R, and BRI information were also collected from the study subjects. BMI was calculated as weight (kg)/height^2^(m^2^); WHtR was calculated as WC (m)/height (m) [16]; WHT.5R was calculated as WC (cm)/height^0.5^ (cm^0.5^) [18]; and BRI was calculated as 364.2−365.5*sqrt (1-(WC (m)/(2π))^2^/(0.5*height (m))^2^) [19] (S1 Table).

### Statistical analysis

All continuous variables were expressed as the mean ± standard deviation (SD) and median (first quartile, third quartile), according to whether they conformed to a normal distribution. Categorical variables were expressed as numbers and frequencies. Chi-square tests were used for the comparison of categorical variables, and the Kruskal-Wallis rank-sum test was used for the comparison of continuous variables. Baseline and last follow-up data were used to represent dynamic changes in anthropometric indicators. Univariate Cox regression models were used to assess the association of demographic and anthropometric indicators (BMI, WC, WHtR, WHT.5R, and BRI) with multimorbidity. Multifactorial Cox regression models were conducted to evaluate the independent effects of two-by-two combinations of BMI, WC, and WHtR and baseline and dynamic changes in BMI, WC, WHtR, WHT.5R, and BRI on the occurrence of multimorbidity. Three Cox regression models were established: the unadjusted model; Model 1 with adjustment for gender, age, and marital status; and Model 2 adjusted for confounders including gender, age, marital status, smoking, alcohol consumption, and physical activity. The proportional hazard assumption was verified with graphical methods. In addition, interaction and stratification analyses were performed to assess potential interactions between WC and demographic indicators and other anthropometric indicators (BMI, WHtR, WHT.5R, and BRI).

The sensitivity analysis was performed in two cases: the first was the populations excluding subjects with one chronic disease at baseline, and the second was included in the model analysis using age stratification. *P* values in this study were all two-tailed and were considered statistically significant when P<0.05. All analyses were performed using SPSS V.21.0 (IBM), and the forest plot was generated by GraphPad Prism 8.

## Results

### Characteristics of the participants

The baseline characteristics of the study subjects with and without multimorbidity are presented in Table 1. Overall, 75,028 subjects with a mean baseline age of 64.83 years were studied, 55.7% of whom were women and 44.3% of whom were men. After a median follow-up of 3.09 years (IQR: 2.17–3.64), multimorbidity occurred in 5,886 participants, including 3,288 women and 2,598 men. The disease composition of these patients with multimorbidity is presented in S2 Table. Subjects who developed multimorbidity had higher levels of BMI, WC, WHtR, WHT.5R, and BRI than those who did not (*P*<0.001). Additionally, those subjects who developed multimorbidity were older and were often physically inactive compared to subjects who did not develop multimorbidity during the follow-up.

**Table 1:** Baseline characteristics of subjects who did and didn’t develop new-onset multimorbidity during follow-up.

	Total (75,028)	Multimorbidity (5,886)	No multimorbidity (69,142)	*p* value
Age (years)	64.83±8.71	67.60±7.41	64.60±8.77	<0.001***
Sex ((n (%))				0.764
Men	33,262 (44.3%)	2,598 (44.1%)	30,664 (44.3%)	
Women	41,766 (55.7%)	3,288 (55.9%)	38,478 (55.7%)	
Marital status ((n (%))				0.020*
Couple	66,249 (88.3%)	5,253 (89.2%)	60,996 (88.2%)	
Single	8,779 (11.7%)	633 (10.8%)	8,146 (11.8%)	
BMI (kg/m^2^)	24.36±2.75	25.19±3.10	24.29±2.71	<0.001***
WC (cm)	83.05±7.65	84.86±8.74	82.90±7.52	<0.001***
WHtR	0.51±0.18	0.52±0.06	0.50±0.05	<0.001***
WHT.5R	6.50±0.63	6.66±0.68	6.49±0.59	<0.001***
BRI	4.54±0.73	4.73±0.83	4.52±0.72	<0.001***
Smoking (n (%))				0.001**
Never	71,816 (95.7%)	5,584 (94.9%)	66,232 (95.8%)	
Current or previous	3,212 (4.3%)	302 (5.1%)	2,910 (4.2%)	
Drinking (n (%))				<0.001***
Former/Never	71,565 (95.4%)	5,531 (94.0%)	66,034 (95.5%)	
Current	3,463 (4.6%)	355 (6.0%)	3,108 (4.5%)	
Physical activity (n (%))				<0.001***
Less than once a week	36,112 (48.1%)	2,242 (38.1%)	33,870 (49.0%)	
More than once a week	38,916 (51.9%)	3,644 (61.9%)	35,272 (51.0%)	

Continuous data are shown as the mean ± SD and categorical data as n (%).

BMI, body mass index; WC, waist circumference; WHtR, waist-to-height ratio; WHT.5R, waist divided by height^0.5^; BRI, body roundness index.

*P-value < 0.05;

**P-value < 0.01;

***P-value < 0.001.

### Association between baseline anthropometric indices and risk of multimorbidity

The relationship between baseline demographic indices and the development of multimorbidity is presented in S3 Table. The results of the Cox proportional hazards regression model indicated that the occurrence of multimorbidity was negatively associated with drinking and physical activity and positively associated with age (*P* <0.001).

**Fig 1:**
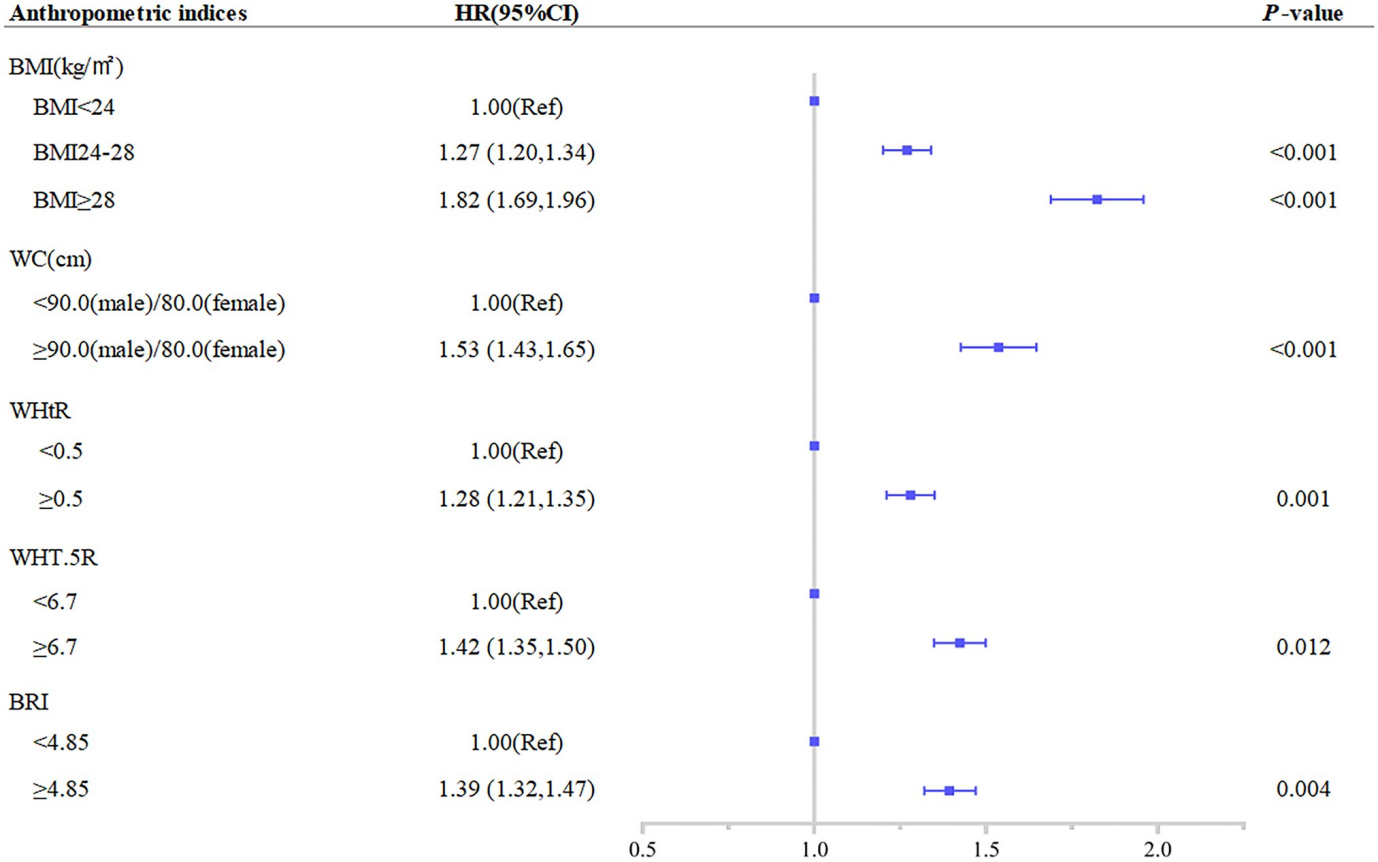
Association between separate anthropometric indices with the development of multimorbidity. (body mass index [BMI], waist circumference [WC], waist-to-height ratio [WHtR], waist divided by height^0.5^ [WHT.5R], body roundness index [BRI]). The correlation was assessed by multivariate cox regression analysis, adjusted by sex, age, marital status, smoking status, drinking status, and physical activity status at baseline. Hazard ratios (HRs) of the anthropometric indices were represented as the squares and 95% confidence intervals (CIs) by the lines through the squares.

Table 2 and Fig 1 presents the HRs and 95% CIs for the association of multimorbidity with the five indicators (BMI, WC, WHtR, WHT.5R, and BRI) at baseline. In the fully adjusted model, those subjects with BMI≥24 (HR 1.27, 95% CI 1.20–1.34) and ≥28 (HR 1.82, 95% CI 1.69–1.96) had a significantly higher risk of multimorbidity with BMI<24 as the reference group. Using internationally accepted diagnostic methods for central obesity, subjects with WC obesity (HR 1.53, 95% CI 1.43–1.65) and WHtR obesity (HR 1.28, 95% CI 1.21–1.35) were at significantly increased risk of multimorbidity compared to those with normal WC and normal WHtR. In addition, the best cutoff value was obtained based on the Youden index for the new anthropometric index, with an increased risk of multimorbidity when WHT.5R≥6.76 (HR 1.42, 95% CI 1.35–1.50) and BRI≥4.84 (HR 1.39, 95% CI 1.32–1.47). Among the above indices, WC was more strongly correlated with the risk of multimorbidity. After full adjustment for sex, age, marriage, drinking status, smoking status, and physical activity status at baseline, elevations in each of the anthropometric measures included in the analysis were independently associated with an increased risk of multimorbidity (Table 2) as follows: BMI (HR 1.23, 95% CI 1.21–1.26); WC (HR 1.18, 95% CI 1.15–1.21); WHtR (HR 1.20, 95% CI 1.17–1.23); WHT.5R (HR 1.19, 95% CI 1.16–1.22); and BRI (HR 1.21, 95% CI 1.18–1.24). Sensitivity analysis revealed that the results remained robust after excluding participants with one chronic condition at baseline and stratified by age. (S4 and S5 Tables).

**Table 2:** Multivariate cox regression models evaluating the associations of baseline anthropometric indices with the development of multimorbidity.

	Unadjusted model		Model 1		Model 2	
	HR (95%CI)	*p* value	HR (95%CI)	*p* value	HR (95%CI)	*p* value
**BMI (kg/m** ^**2**^ **)**						
As continuous variables (per SD increment)	1.24 (1.21,1.27)	<0.001***	1.24 (1.21,1.27)	<0.001***	1.23 (1.21,1.26)	<0.001***
<24.0	1.0		1.0		1.0	
24.0–28.0	1.27 (1.20,1.34)	<0.001***	1.28 (1.21,1.35)	<0.001***	1.27 (1.20,1.34)	<0.001***
≥28.0	1.86 (1.73,2.01)	<0.001***	1.85 (1.72,2.00)	<0.001***	1.82 (1.69,1.96)	<0.001***
**WC (cm)**						
As continuous variables (per SD increment)	1.20 (1.17,1.22)	<0.001***	1.19 (1.16,1.22)	<0.001***	1.18 (1.15,1.21)	<0.001***
<90 in males or <80 in females	1.0		1.0		1.0	
≥90 in males or ≥80 in females	1.53 (1.43,1.64)	<0.001***	1.54 (1.43,1.65)	<0.001***	1.53 (1.43,1.65)	<0.001***
**WHtR**						
As continuous variables (per SD increment)	1.23 (1.20,1.26)	<0.001***	1.21 (1.18,1.23)	<0.001***	1.20 (1.17,1.23)	<0.001***
<0.5	1.0		1.0		1.0	
≥0.5	1.35 (1.28,1.42)	<0.001***	1.30 (1.23,1.37)	<0.001***	1.28 (1.21,1.35)	0.001**
**WHT.5R**						
As continuous variables (per SD increment)	1.22 (1.19,1.25)	<0.001***	1.20 (1.17,1.23)	<0.001***	1.19 (1.16,1.22)	<0.001***
<6.76	1.0		1.0		1.0	
≥6.76	1.50 (1.42,1.58)	<0.001***	1.44 (1.37,1.52)	<0.001***	1.42 (1.35,1.50)	<0.001***
**BRI**						
As continuous variables (per SD increment)	1.22 (1.19,1.25)	<0.001***	1.22 (1.18,1.25)	<0.001***	1.21 (1.18,1.24)	<0.001***
<4.84	1.0		1.0		1.0	
≥4.84	1.45 (1.38,1.53)	<0.001***	1.42 (1.34,1.50)	<0.001***	1.39 (1.32,1.47)	<0.001***

BMI, body mass index; WC, waist circumference; WHtR, waist-to-height ratio; WHT.5R, waist divided by height^0.5^; BRI, body roundness index.

Model 1: adjusted by sex, age, marital status.

Model 2: adjusted by sex, age, marital status, smoking status, drinking status, physical activity.

*P-value < 0.05;

**P-value < 0.01;

***P-value < 0.001.

**Fig 2:**
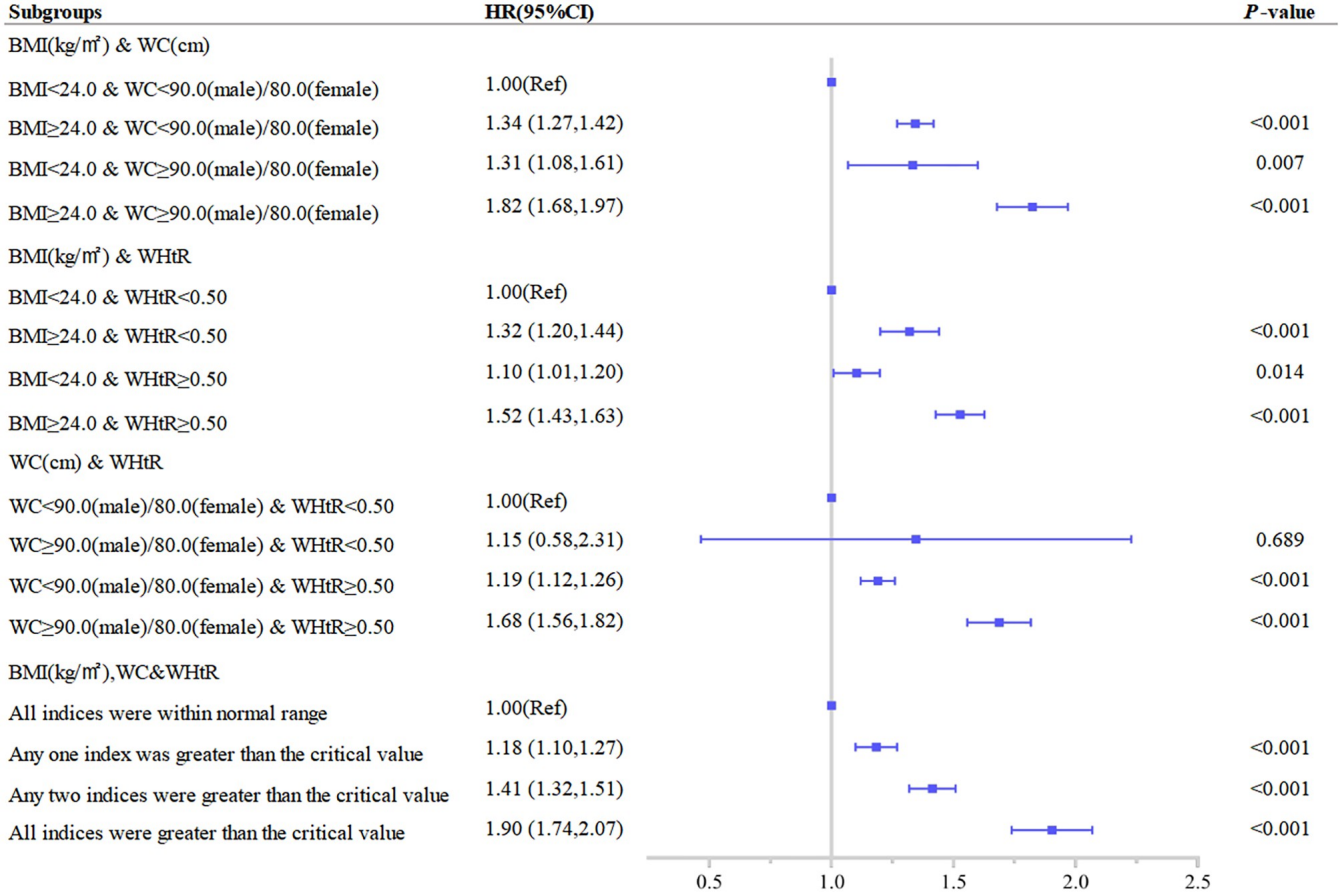
Association between different combinations of body mass index (BMI) and established anthropometric indices of central obesity(waist circumference [WC], waist-to-height ratio [WHtR]) with the development of multimorbidity. The correlation was assessed by multivariate cox regression analysis, adjusted by sex, age, marital status, smoking status, drinking status, and physical activity status at baseline. Hazard ratios (HRs) of the anthropometric indices were represented as the squares and 95% confidence intervals (CIs) by the lines through the squares.

As presented in Table 3, the risk of new-onset multimorbidity was significantly increased at baseline for men with WC≥90 cm or women with WC≥80 cm, regardless of whether BMI was within normal limits (BMI<24kg/m^2^), and elevated baseline WC (WC≥90 cm in men or WC≥80 cm in women) was associated with an increased risk of multimorbidity with baseline BMI<24kg/m^2^ (HR 1.31, 95% CI 1.08–1.61). The risk of multimorbidity increased further when BMI≥24kg/m^2^ (HR 1.82, 95% CI 1.68–1.97) at baseline. The highest risk of multimorbidity (HR 1.90, 95% CI 1.74–2.07) was observed when BMI, WC, and WHtR were all elevated and considered to be abnormal (Fig 2). Stratified analysis and interaction results revealed that WC did not interact significantly with other statistical variables (S6 Table).

**Table 3:** Multivariate cox regression models evaluating the associations of different combinations of BMI, WC, and WHtR with the development of multimorbidity.

	Unadjusted model		Model 1		Model 2	
	HR (95%CI)	*p* value	HR (95%CI)	*p* value	HR (95%CI)	*p* value
**BMI (kg/m** ^**2**^ **) & WC (cm)**						
BMI<24 & WC<90 (male)/80 (female)	1.0		1.0		1.0	
BMI≥24 & WC<90 (male)/80 (female)	1.36 (1.29,1.44)	<0.001***	1.36 (1.29,1.44)	<0.001***	1.34 (1.27,1.42)	<0.001***
BMI<24 & WC≥90 (male)/80 (female)	1.33 (1.09,1.62)	0.006**	1.32 (1.08,1.61)	0.007**	1.31 (1.08,1.61)	0.007**
BMI≥24 & WC≥90 (male)/80 (female)	1.87 (1.73,2.02)	<0.001***	1.86 (1.72,2.01)	<0.001***	1.82 (1.68,1.97)	<0.001***
**BMI (kg/m** ^**2**^ **) & WHtR**						
BMI<24 & WHtR<0.5	1.0		1.0		1.0	
BMI≥24 & WHtR<0.5	1.30 (1.19,1.43)	<0.001***	1.33 (1.21,1.45)	<0.001***	1.32 (1.20,1.44)	<0.001***
BMI<24 & WHtR≥0.5	1.16 (1.07,1.26)	<0.001***	1.11 (1.02,1.20)	0.008**	1.10 (1.01,1.20)	0.014*
BMI≥24 & WHtR≥0.5	1.61 (1.51,1.72)	<0.001***	1.56 (1.46,1.67)	<0.001***	1.52 (1.43,1.63)	<0.001***
**WC (cm) & WHtR**						
WC<90 (male)/80 (female) & WHtR<0.5	1.0		1.0		1.0	
WC≥90 (male)/80 (female) & WHtR<0.5	1.09 (0.54,2.18)	0.812	1.18 (0.59,2.37)	0.636	1.15 (0.58,2.31)	0.689
WC<90 (male)/80 (female) & WHtR≥0.5	1.25 (1.18,1.32)	<0.001***	1.21 (1.14,1.28)	<0.001***	1.19 (1.12,1.26)	<0.001***
WC≥90 (male)/80 (female) & WHtR≥0.5	1.73 (1.60,1.86)	<0.001***	1.72 (1.59,1.86)	<0.001***	1.68 (1.56,1.82)	<0.001***
**BMI, WC & WHtR**						
All indicators were normal	1.0		1.0		1.0	
Any one indicator was abnormal	1.21 (1.12,1.30)	<0.001***	1.19 (1.10,1.28)	<0.001***	1.18 (1.10,1.27)	<0.001***
Any two indicators were abnormal	1.48 (1.38,1.59)	<0.001***	1.44 (1.35,1.55)	<0.001***	1.41 (1.32,1.51)	<0.001***
All indicators were abnormal	1.96 (1.80,2.13)	<0.001***	1.94 (1.78,2.12)	<0.001***	1.90 (1.74,2.07)	<0.001***

BMI, body mass index; WC, waist circumference; WHtR, waist-to-height ratio; WHT.5R, waist divided by height^0.5^; BRI, body roundness index.

Model 1: adjusted by sex, age, marital status.

Model 2: adjusted by sex, age, marital status, smoking status, drinking status, physical activity.

*P-value < 0.05;

**P-value < 0.01;

***P-value < 0.001.

### Association between dynamic changes in anthropometric indices and risk of multimorbidity

**Fig 3:**
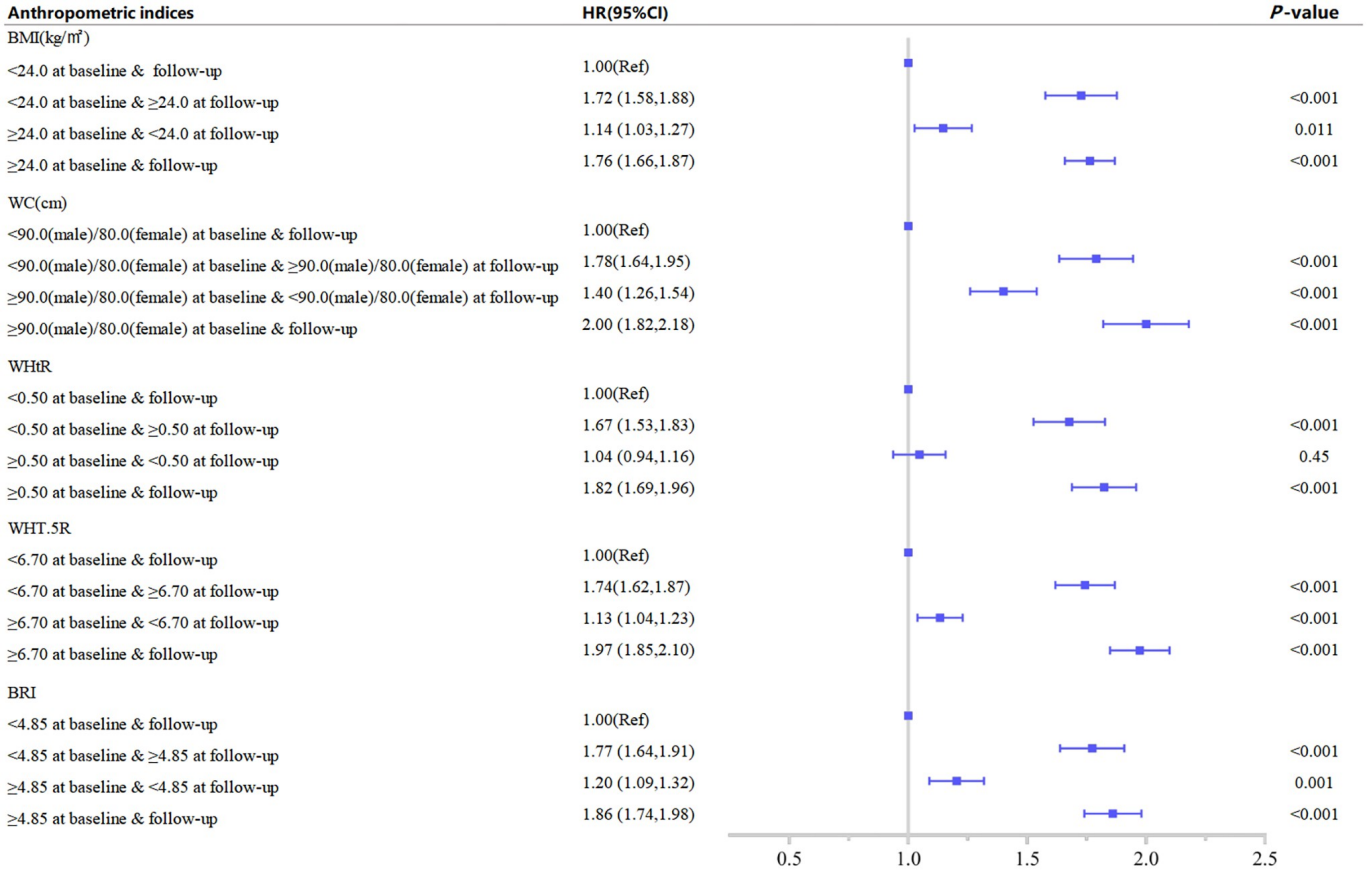
Association between dynamic changes of separate anthropometric indices with the development of multimorbidity. (body mass index [BMI], waist circumference [WC], waist-to-height ratio [WHtR], waist divided by height^0.5^ [WHT.5R], body roundness index [BRI]). The correlation was assessed by multivariate cox regression analysis, adjusted by sex, age, marital status, smoking status, drinking status, and physical activity status at baseline. Hazard ratios (HRs) of the anthropometric indices were represented as the squares and 95% confidence intervals (CIs) by the lines through the squares.

Table 4 and Fig 3 indicated that subjects with elevated WC at baseline or follow-up (WC≥90 cm in men or WC≥80 cm in women) exhibited a higher risk of multimorbidity than subjects with normal WC (WC<90 cm in men or WC<80 cm in women) at baseline and follow-up in the fully adjusted Model 2. When elevated WC was identified at baseline, the risk of multimorbidity at follow-up (HR 2.00, 95% CI 1.82–2.18) was significantly higher in subjects with elevated WC than in subjects whose WC had recovered to below the critical value (HR 1.40, 95% CI 1.26–1.54). Among subjects with BMI in the normal range at baseline, those with BMI≥24 kg/m^2^ during follow-up had a significantly increased risk of multimorbidity compared with those who maintained BMI<24 kg/m^2^ at follow-up. The same was observed in WHtR, WHT.5R, and BRI.

**Table 4:** Multivariate cox regression models evaluating the associations of dynamic changes of established anthropometric indices with the development of multimorbidity.

	Unadjusted model		Model 1		Model 2	
	HR (95%CI)	*p* value	HR (95%CI)	*p* value	HR (95%CI)	*p* value
**Dynamic changes of BMI (kg/m** ^**2**^ **)**						
<24 at baseline & <24 at follow-up	1.0		1.0		1.0	
<24 at baseline & ≥24 at follow-up	1.76 (1.61,1.92)	<0.001***	1.71 (1.57,1.87)	<0.001***	1.72 (1.58,1.88)	<0.001***
≥24 at baseline & <24 at follow-up	1.16 (1.05,1.29)	0.005**	1.15 (1.04,1.27)	0.009**	1.14 (1.03,1.27)	0.011*
≥24 at baseline & ≥24 at follow-up	1.81 (1.70,1.92)	<0.001***	1.79 (1.69,1.91)	<0.001***	1.76 (1.66,1.87)	<0.001***
**Dynamic changes of WC (cm)**						
<90(male)/80(female) at baseline & <90(male)/80(female)at follow-up	1.0		1.0		1.0	
<90(male)/80(female) at baseline & ≥90(male)/80(female)at follow-up	1.70 (1.56,1.85)	<0.001***	1.77 (1.63,1.94)	<0.001***	1.78 (1.64,1.95)	<0.001***
≥90(male)/80(female) at baseline & <90(male)/80(female)at follow-up	1.33 (1.21,1.47)	<0.001***	1.41 (1.28,1.56)	<0.001***	1.40 (1.26,1.54)	<0.001***
≥90(male)/80(female) at baseline & ≥90(male)/80(female)at follow-up	1.94 (1.78,2.11)	<0.001***	2.03 (1.85,2.22)	<0.001***	2.00 (1.82,2.18)	<0.001***
**Dynamic changes of WHtR**						
<0.5 at baseline & <0.5 at follow-up	1.0				1.0	
<0.5 at baseline & ≥0.5 at follow-up	1.70 (1.56,1.85)	<0.001***	1.66 (1.52,1.82)	<0.001***	1.67 (1.53,1.83)	<0.001***
≥0.5 at baseline & <0.5 at follow-up	1.05 (0.95,1.17)	0.348	1.05 (0.94,1.16)	0.417	1.04 (0.94,1.16)	0.450
≥0.5 at baseline & ≥0.5 at follow-up	1.93 (1.79,2.07)	<0.001***	1.85 (1.72,1.99)	<0.001***	1.82 (1.69,1.96)	<0.001***
**Dynamic changes of WHT.5R**						
<6.76 at baseline & <6.76 at follow-up	1.0		1.0		1.0	
<6.76 at baseline & ≥6.76 at follow-up	1.79 (1.67,1.92)	<0.001***	1.74 (1.62,1.86)	<0.001***	1.74 (1.62,1.87)	<0.001***
≥6.76 at baseline & <6.76 at follow-up	1.15 (1.05,1.25)	<0.001***	1.13 (1.04,1.23)	<0.001***	1.13 (1.04,1.23)	<0.001***
≥6.76 at baseline & ≥6.76 at follow-up	2.13 (2.00,2.26)	<0.001***	2.00 (1.87,2.13)	<0.001***	1.97 (1.85,2.10)	<0.001***
**Dynamic changes of BRI**						
<4.84 at baseline & <4.84 at follow-up	1.0		1.0		1.0	
<4.84 at baseline & ≥4.84 at follow-up	1.74 (1.62,1.88)	<0.001***	1.77 (1.64,1.91)	<0.001***	1.77 (1.64,1.91)	<0.001***
≥4.84 at baseline & <4.84 at follow-up	1.18 (1.08,1.30)	0.003**	1.22 (1.11,1.34)	<0.001***	1.20 (1.09,1.32)	0.001**
≥4.84 at baseline & ≥4.84 at follow-up	1.86 (1.75,1.97)	<0.001***	1.89 (1.77,2.02)	<0.001***	1.86 (1.74,1.98)	<0.001***

BMI, body mass index; WC, waist circumference; WHtR, waist-to-height ratio; WHT.5R, waist divided by height^0.5^; BRI, body roundness index.

Model 1: adjusted by sex, age, and marital status.

Model 2: adjusted by sex, age, marital status, smoking status, drinking status, and physical activity.

*P-value < 0.05;

**P-value < 0.01;

***P-value < 0.001.

## Discussion

In the current retrospective cohort of middle-aged and older adults, our data suggest that the anthropometric parameters analyzed in this study, namely, BMI, WC, WHtR, WHT.5R, and BRI, are independently associated with an increased risk of multimorbidity, and of the above five indicators, WC had the strongest association with the risk of multimorbidity. The dynamics of the indices also revealed a stronger correlation between WC and the risk of multimorbidity than BMI, WHtR, WHT.5R, and BRI after controlling for the various confounding factors. In addition, elevated WC at baseline was positively associated with an increased risk of multimorbidity, regardless of whether BMI was within the normal range.

Our study supports the findings of previous studies that general obesity is positively associated with the risk of multimorbidity [29, 30] and provides further evidence that abdominal obesity is associated with an increased risk of multimorbidity [31] and suggests that WC may be a better screening indicator for multimorbidity than BMI, WHtR, WHT.5R, and BRI. Data from the U.S. National Health and Nutrition Examination Survey study (3,652 men and 3,609 women) reported that elevated BMI is strongly associated with the risk of multimorbidity in people aged ≥60 years [29]. And a cross-sectional study of 5,493 subjects aged 65 years or older from China indicated that WC and BMI are strongly associated with the risk of multimorbidity and strongly suggested that WC may be a better indicator for chronic disease screening than BMI [31]. Furthermore, in previous Western and Asian population studies, WC, as the primary measure of abdominal obesity, has been widely used as an indicator of obesity-related health risks [32, 33]. Some studies have demonstrated that WC is a stronger predictor of cardiometabolic disease risk than BMI [34, 35], and it has been suggested that WC may play an important role in the early development of metabolic syndrome [36].

Our data suggest that for the middle-aged and elderly populations, normal weight but central obesity was also associated with a risk of multimorbidity. This finding is supported by a previous study that concluded people with normal BMI but abdominal obesity are equally likely to have multiple noncommunicable diseases [14]. This suggests that abdominal obesity is a risk factor that is strongly associated with multimorbidity, albeit it is easily overlooked compared to generalized obesity as measured by BMI. Several mechanisms can be used to explain our findings. To begin with, abdominal obesity predisposes the body to significantly elevated concentrations of plasma triglycerides, low-density lipoproteins, and very-low-density lipoproteins, and elevated concentrations of all three have been shown to increase the risk of hyperlipidemia, insulin resistance, cardiovascular disease, and hypertension [14]. Additionally, people with normal weight but central obesity tend to have excess visceral fat, which, according to a previous study, may result in high doses of adipokines from the portal vein to the liver and other body tissues [37]. Moreover, these adipokines have serious implications for noncommunicable diseases such as diabetes [38], hypertension [39], heart disease [40], kidney disease [41], cancer, and other health problems [42]. Therefore, in addition to BMI, the central obesity index WC can be measured to determine whether patients of normal weight but with central obesity are at risk for multimorbidity, thus providing incremental benefits for screening the middle-aged and elderly populations at high risk for multimorbidity.

Many previous studies have indicated that WHT.5R and BRI are more strongly associated with the risk of cardiovascular disease and metabolic syndrome than BMI, WC, or WHtR [18, 20, 21], but it is unclear whether there is an association between WHT.5R and BRI and the risk of multimorbidity. Our results demonstrate that WHT.5R and BRI are independently and positively associated with the risk of multimorbidity, and both are more strongly associated with multimorbidity than BMI and WHtR but weaker than WC, suggesting that WHT.5R and BRI are better than BMI and WHtR in screening for multimorbidity. More rigorously designed cohort studies must be conducted to further support our results.

Our study also reveals a tendency to reduce the risk of multimorbidity when WC, WHtR, WHT.5R, and BRI are reversed from abnormal to normal levels. Additionally, an increase in the risk of multimorbidity was observed with an increase in WC during follow-up. It has been well documented that lifestyle interventions, and dietary modifications including fruit and vegetable intake, weight loss, and moderate-to-vigorous exercise reduce the risk of developing many diseases, such as diabetes, cardiovascular disease, and cancer [43–45]. Therefore, long-term monitoring of these anthropometric indicators and the application of timely lifestyle interventions are expected to facilitate the transformation of these obesity indices from abnormal to normal levels, which is important for preventing or delaying the onset of multimorbidity.

Our study has several strengths. First, we are the first cohort study to explore the association of central obesity indicators with the risk of developing multimorbidity, bridging the limitations of previous multimorbidity cross-sectional studies. Second, the identification of chronic diseases was relatively accurate and comprehensive, including those self-reported by participants and diagnoses made by physicians based on a comprehensive professional examination. Finally, in addition to baseline values of anthropometric indices, we also examined the associations between trends in these indices and different combinations of BMI, WC, and WHtR and the risk of multimorbidity, thus providing more insight into the impact of central obesity on multimorbidity.

However, our study has some limitations. First, some important diseases, such as kidney disease, were not included in the analysis due to a lack of data, which may lead to an underestimation of the risk of developing multimorbidity. Second, even though the major confounding factors were adjusted in the analysis, the effects of some confounding factors still existed, including unmeasured factors such as economic income level and dietary habits. Third, our subjects were all from the Jinshui community and thus are not fully representative of the entire Chinese population. Finally, the retrospective nature of the study prevents us from accounting for those with missing data and those lost to follow-up.

## Conclusion

Our study suggests that central obesity is an important independent and modifiable risk factor for multimorbidity in middle-aged and elderly populations. Measuring central obesity indices, especially WC, in addition to BMI in health screenings may provide incremental benefits for multimorbidity screening and prevention of multimorbidity issues in the middle-aged and elderly populations.

## Supporting information

S1 Fig Flow diagram of participant selection. (PDF)

S1 Table Formulas of anthropometric indices. (DOCX)

S2 Table Disease composition of new-onset multimorbidity. (DOCX)

S3 Table Univariate cox regression models evaluating the association of demographic, and anthropometric indexes with multimorbidity. (DOCX)

S4 Table Risk of multimorbidity in participants without chronic disease at baseline. (DOCX)

S5 Table Risk of multimorbidity in participants after stratification according to age. (DOCX)

S6 Table Association between WC and multimorbidity according to baseline characteristics. (DOCX)
