# Supplementary figures and images for: Association of anthropometric indices with the development of multimorbidity in middle-aged and older adults: A retrospective cohort study
Source: PLoS One. 2022 Oct 14;17(10):e0276216. doi: 10.1371/journal.pone.0276216 (PMC9565419; doi:10.1371/journal.pone.0276216)

**S1 Figure** Flow diagram of participant selection

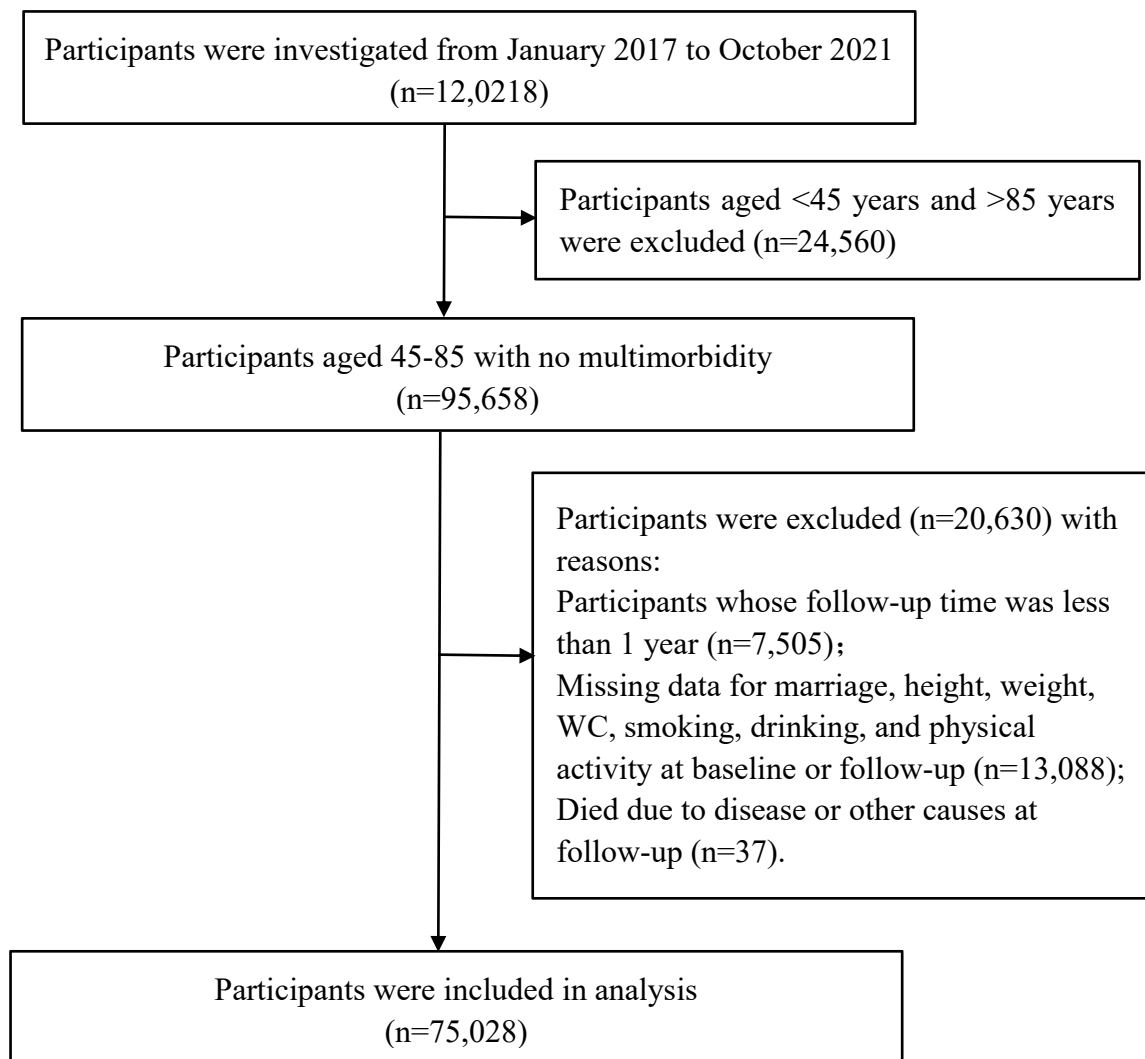

Supplement: S1 Fig — (PDF) [file pone.0276216.s001.pdf]
