# Supplementary material for: Association of anthropometric indices with the development of multimorbidity in middle-aged and older adults: A retrospective cohort study
Source: PLoS One. 2022 Oct 14;17(10):e0276216. doi: 10.1371/journal.pone.0276216 (PMC9565419; doi:10.1371/journal.pone.0276216)
Supplement: S1 Table — (DOCX) [file pone.0276216.s002.docx]

| **S1 Table** Formulas of anthropometric indices. | |
| --- | --- |
| **Anthropometric indices** | **Formula** |
| Body mass index (BMI) |  |
| waist-to-height ratio (WHtR) |  |
| waist divided by height0.5 (WHT.5R) |  |
| Body roundness index (BRI) |  |
| Abbreviations: BMI, body mass index; WC, waist circumference; WHtR,waist-to-height ratio; WHT.5R, waist divided by height^0.5^; BRI, body roundness index. | |
|  |  |
