# Supplementary material for: Association of anthropometric indices with the development of multimorbidity in middle-aged and older adults: A retrospective cohort study
Source: PLoS One. 2022 Oct 14;17(10):e0276216. doi: 10.1371/journal.pone.0276216 (PMC9565419; doi:10.1371/journal.pone.0276216)
Supplement: S2 Table — (DOCX) [file pone.0276216.s003.docx]

| **S2 Table** Disease composition of new-onset multimorbidity. | | | |
| --- | --- | --- | --- |
| **Chronic condition** | Total **(5886)** | **Female (3288)** | **Male (2598)** |
| Hypertension | 5643 (95.8%) | 3161 (96.1%) | 2482 (95.5%) |
| Diabetes | 3978 (67.5%) | 2261 (68.7%) | 1717 (66.0%) |
| Heart disease | 1762 (29.9%) | 958 (29.1%) | 804 (30.9%) |
| Stroke | 534 (9.0%) | 261 (7.9%) | 273 (10.5%) |
| Cancer | 34 (0.5%) | 19 (0.5%) | 15 (0.5%) |
| Chronic obstructive pulmonary disease | 14 (0.2%) | 5 (0.1%) | 9 (0.3%) |
| Psychiatric problems | 19 (0.3%) | 10 (0.3%) | 9 (0.3%) |
|  | | | |
