# Supplementary material for: Association of anthropometric indices with the development of multimorbidity in middle-aged and older adults: A retrospective cohort study
Source: PLoS One. 2022 Oct 14;17(10):e0276216. doi: 10.1371/journal.pone.0276216 (PMC9565419; doi:10.1371/journal.pone.0276216)
Supplement: S3 Table — (DOCX) [file pone.0276216.s004.docx]

| **S3 Table**  Univariate cox regression models evaluating the association of demographic, and anthropometric indexes with multimorbidity. | | |
| --- | --- | --- |
|  | **HR, 95% CI** | **P-value** |
| Age (years) |  |  |
| 45-59 | 1 | - |
| 60-74 | 2.44 (2.24,2.65) | <0.001^***^ |
| 75-85 | 2.86 (2.60,3.15) | <0.001^***^ |
| Sex (n (%)) |  |  |
| Male | 1 | - |
| Female | 0.99 (0.95,1.05) | 0.928 |
| Marital status |  |  |
| Single | 1 | - |
| Couple | 1.00 (0.92,1.09) | 0.960 |
| BMI (kg/m^2^) |  |  |
| <24 | 1 | - |
| 24.0-28.0 | 1.27 (1.20,1.34) | <0.001^***^ |
| ≥28 | 1.86 (1.73,2.01) | <0.001^***^ |
| WC (cm) |  |  |
| <90 in male or <80 in female | 1 | - |
| ≥90 in male or ≥80 in female | 1.53 (1.43,1.64) | <0.001^***^ |
| WHtR |  |  |
| <0.5 | 1 | - |
| ≥0.5 | 1.35 (1.28,1.42) | <0.001^***^ |
| WHT.5R |  |  |
| <6.76 | 1 | - |
| ≥6.76 | 1.50 (1.42,1.58) | <0.001^***^ |
| BRI |  |  |
| <4.84 | 1 | - |
| ≥4.84 | 1.45 (1.38,1.53) | <0.001^***^ |
| Smoking (n (%)) |  |  |
| No | 1 | - |
| Yes | 1.11 (0.99,1.25) | 0.070 |
| Drinking (n (%)) |  |  |
| No | 1 | - |
| Yes | 1.24 (1.11,1.38) | <0.001^***^ |
| Physical activity (n (%)) |  |  |
| Yes | 1 | - |
| No | 1.30 (1.23,1.37) | <0.001^***^ |
| Abbreviations: BMI, body mass index; WC, waist circumference; WHtR, waist-to-height ratio; WHT.5R, waist divided by height^0.5^; BRI, body roundness index.  ^*^*P*-value < 0.05; ^**^*P*-value < 0.01; ^***^*P*-value < 0.001. | | |
