# Supplementary material for: Association of anthropometric indices with the development of multimorbidity in middle-aged and older adults: A retrospective cohort study
Source: PLoS One. 2022 Oct 14;17(10):e0276216. doi: 10.1371/journal.pone.0276216 (PMC9565419; doi:10.1371/journal.pone.0276216)
Supplement: S4 Table — (DOCX) [file pone.0276216.s005.docx]

| **S4 Table** Risk of multimorbidity in participants without chronic disease at baseline. | | | | | | | | |
| --- | --- | --- | --- | --- | --- | --- | --- | --- |
|  | \| **Unadjusted model** \| \| --- \| | |  | \| **Model 1** \| \| --- \| | |  | \| **Model 2** \| \| --- \| | |
|  | **HR (95%CI)** | ***P*-value** |  | **HR (95%CI)** | ***P*-value** |  | **HR (95%CI)** | ***P*-value** |
| **BMI (kg/m^2^)** |  |  |  |  |  |  |  |  |
| As continuous variables (per SD increment) | 1.34 (1.26,1.43) | <0.001^***^ |  | 1.33 (1.25,1.42) | <0.001^***^ |  | 1.33 (1.25,1.42) | <0.001^***^ |
| <24.0 | 1.0 |  |  | 1.0 |  |  | 1.0 |  |
| 24.0-28.0 | 1.39 (1.18,1.65) | <0.001^***^ |  | 1.38 (1.17,1.63) | <0.001^***^ |  | 1.38 (1.17,1.64) | <0.001^***^ |
| ≥28.0 | 2.13 (1.68,2.71) | <0.001^***^ |  | 2.12 (1.67,2.69) | <0.001^***^ |  | 2.15 (1.69,2.73) | <0.001^***^ |
| **WC (cm)** |  |  |  |  |  |  |  |  |
| As continuous variables (per SD increment) | 1.17 (1.09,1.25) | <0.001^***^ |  | 1.14 (1.06,1.23) | <0.001^***^ |  | 1.15 (1.07,1.24) | <0.001^***^ |
| <90 in males or <80 in females | 1.0 |  |  | 1.0 |  |  | 1.0 |  |
| ≥90 in males or ≥80 in females | 1.51 (1.29,1.77) | <0.001^***^ |  | 1.69 (1.42,2.01) | <0.001^***^ |  | 1.68 (1.41,2.01) | <0.001^***^ |
| **WHtR** |  |  |  |  |  |  |  |  |
| As continuous variables (per SD increment) | 1.15 (1.07,1.24) | <0.001^***^ |  | 1.15 (1.06,1.23) | <0.001^***^ |  | 1.15 (1.07,1.24) | <0.001^***^ |
| <0.5 | 1.0 |  |  | 1.0 |  |  | 1.0 |  |
| ≥0.5 | 1.43 (1.22,1.68) | <0.001^***^ |  | 1.40 (1.18,1.64) | <0.001^***^ |  | 1.38 (1.17,1.63) | <0.001^***^ |
| **WHT.5R** |  |  |  |  |  |  |  |  |
| As continuous variables (per SD increment) | 1.22 (1.14,1.31) | <0.001^***^ |  | 1.19 (1.11,1.28) | <0.001^***^ |  | 1.19 (1.10,1.28) | <0.001^***^ |
| <6.33 | 1.0 |  |  | 1.0 |  |  | 1.0 |  |
| ≥6.33 | 1.50 (1.28,1.77) | <0.001^***^ |  | 1.45 (1.23,1.72) | <0.001^***^ |  | 1.46 (1.24,1.73) | <0.001^***^ |
| **BRI** |  |  |  |  |  |  |  |  |
| As continuous variables (per SD increment) | 1.18 (1.09,1.26) | <0.001^***^ |  | 1.21 (1.12,1.31) | <0.001^***^ |  | 1.21 (1.11,1.31) | <0.001^***^ |
| <4.50 | 1.0 |  |  | 1.0 |  |  | 1.0 |  |
| ≥4.50 | 1.32 (1.13,1.54) | 0.001^**^ |  | 1.38 (1.16,1.65) | <0.001^***^ |  | 1.37 (1.16,1.64) | <0.001^***^ |
| BMI, body mass index; WC, waist circumference; WHtR, waist-to-height ratio; WHT.5R, waist divided by height^0.5^; BRI, body roundness index.  Model 1: adjusted by sex, age, Marital status.  Model 2: adjusted by sex, age, Marital status, smoking status, drinking status,Physical activity.  ^*^*P*-value < 0.05; ^**^*P*-value < 0.01; ^***^*P*-value < 0.001. | | | | | | | | |
