# Supplementary material for: Association of anthropometric indices with the development of multimorbidity in middle-aged and older adults: A retrospective cohort study
Source: PLoS One. 2022 Oct 14;17(10):e0276216. doi: 10.1371/journal.pone.0276216 (PMC9565419; doi:10.1371/journal.pone.0276216)
Supplement: S5 Table — (DOCX) [file pone.0276216.s006.docx]

| **S5 Table** Risk of multimorbidity in participants after stratification according to age. | | | | | | | | |
| --- | --- | --- | --- | --- | --- | --- | --- | --- |
|  | \| **Unadjusted model** \| \| --- \| | |  | \| **Model 1** \| \| --- \| | |  | \| **Model 2** \| \| --- \| | |
|  | **HR (95%CI)** | ***P*-value** |  | **HR (95%CI)** | ***P*-value** |  | **HR (95%CI)** | ***P*-value** |
| **Middle-aged adults (45-64years)** | | | | | | | | |
| BMI (kg/m^2^) |  |  |  |  |  |  |  |  |
| <24.0 | 1.0 |  |  | 1.0 |  |  | 1.0 |  |
| 24.0-28.0 | 1.63 (1.37,1.94) | <0.001^***^ |  | 1.58 (1.33,1.87) | <0.001^***^ |  | 1.43 (1.21,1.71) | <0.001^***^ |
| ≥28.0 | 2.88 (1.68,2.71) | <0.001^***^ |  | 2.82 (2.26,3.52) | <0.001^***^ |  | 2.46 (1.97,3.08) | <0.001^***^ |
| WC (cm) |  |  |  |  |  |  |  |  |
| <90 in males or <80 in females | 1.0 |  |  | 1.0 |  |  | 1.0 |  |
| ≥90 in males or ≥80 in females | 2.00 (1.63,2.46) | <0.001^***^ |  | 1.99 (1.61,2.48) | <0.001^***^ |  | 1.87 (1.51,2.32) | <0.001^***^ |
| WHtR |  |  |  |  |  |  |  |  |
| <0.5 | 1.0 |  |  | 1.0 |  |  | 1.0 |  |
| ≥0.5 | 1.64 (1.40,1.92) | <0.001^***^ |  | 1.57 (1.34,1.84) | <0.001^***^ |  | 1.44 (1.22,1.69) | <0.001^***^ |
| WHT.5R |  |  |  |  |  |  |  |  |
| <6.33 | 1.0 |  |  | 1.0 |  |  | 1.0 |  |
| ≥6.33 | 1.75 (1.49,2.06) | <0.001^***^ |  | 1.71 (1.45,2.01) | <0.001^***^ |  | 1.58 (1.34,1.86) | <0.001^***^ |
| BRI |  |  |  |  |  |  |  |  |
| <4.50 | 1.0 |  |  | 1.0 |  |  | 1.0 |  |
| ≥4.50 | 1.68 (1.42,1.99) | <0.001^***^ |  | 1.64 (1.37,1.96) | <0.001^***^ |  | 1.51 (1.26,1.80) | <0.001^***^ |
| **Younger older adults (65-74 years)** | | | | | | | | |
| BMI (kg/m^2^) |  |  |  |  |  |  |  |  |
| <24.0 | 1.0 |  |  | 1.0 |  |  | 1.0 |  |
| 24.0-28.0 | 1.38 (1.29,1.48) | <0.001^***^ |  | 1.38 (1.29,1.48) | <0.001^***^ |  | 1.36 (1.27,1.46) | <0.001^***^ |
| ≥28.0 | 2.16 (1.98,2.36) | <0.001^***^ |  | 2.15 (1.97,2.34) | <0.001^***^ |  | 2.09 (1.92,2.29) | <0.001^***^ |
| WC (cm) |  |  |  |  |  |  |  |  |
| <90 in males or <80 in females | 1.0 |  |  | 1.0 |  |  | 1.0 |  |
| ≥90 in males or ≥80 in females | 1.52 (1.40,1.64) | <0.001^***^ |  | 1.59 (1.46,1.72) | <0.001^***^ |  | 1.57 (1.44,1.70) | <0.001^***^ |
| WHtR |  |  |  |  |  |  |  |  |
| <0.5 | 1.0 |  |  | 1.0 |  |  | 1.0 |  |
| ≥0.5 | 1.31 (1.26,1.39) | <0.001^***^ |  | 1.31 (1.23,1.40) | <0.001^***^ |  | 1.29 (1.21,1.38) | <0.001^***^ |
| WHT.5R |  |  |  |  |  |  |  |  |
| <6.33 | 1.0 |  |  | 1.0 |  |  | 1.0 |  |
| ≥6.33 | 1.51 (1.42,1.61) | <0.001^***^ |  | 1.49 (1.40,1.59) | <0.001^***^ |  | 1.47 (1.38,1.56) | <0.001^***^ |
| BRI |  |  |  |  |  |  |  |  |
| <4.50 | 1.0 |  |  | 1.0 |  |  | 1.0 |  |
| ≥4.50 | 1.39 (1.30,1.48) | 0.001^**^ |  | 1.44 (1.35,1.54) | <0.001^***^ |  | 1.42 (1.33,1.52) | <0.001^***^ |
| **Older adults (75-85 years)** | | | | | | | | |
| BMI (kg/m^2^) |  |  |  |  |  |  |  |  |
| <24.0 | 1.0 |  |  | 1.0 |  |  | 1.0 |  |
| 24.0-28.0 | 1.24 (1.09,1.40) | 0.001^**^ |  | 1.23 (1.08,1.40) | 0.002^**^ |  | 1.21 (1.07,1.38) | 0.003^**^ |
| ≥28.0 | 1.94 (1.62,2.32) | <0.001^***^ |  | 1.92 (1.60,2.29) | <0.001^***^ |  | 1.87 (1.56,2.24) | <0.001^***^ |
| WC (cm) |  |  |  |  |  |  |  |  |
| <90 in males or <80 in females | 1.0 |  |  | 1.0 |  |  | 1.0 |  |
| ≥90 in males or ≥80 in females | 1.40 (1.20,1.64) | <0.001^***^ |  | 1.42 (1.20,1.67) | <0.001^***^ |  | 1.39 (1.18,1.64) | <0.001^***^ |
| WHtR |  |  |  |  |  |  |  |  |
| <0.5 | 1.0 |  |  | 1.0 |  |  | 1.0 |  |
| ≥0.5 | 1.24 (1.10,1.40) | 0.001^**^ |  | 1.24 (1.10,1.41) | 0.001^**^ |  | 1.21 (1.07,1.38) | 0.003^**^ |
| WHT.5R |  |  |  |  |  |  |  |  |
| <6.33 | 1.0 |  |  | 1.0 |  |  | 1.0 |  |
| ≥6.33 | 1.44 (1.28,1.63) | <0.001^***^ |  | 1.43 (1.27,1.62) | <0.001^***^ |  | 1.40 (1.24,1.58) | <0.001^***^ |
| BRI |  |  |  |  |  |  |  |  |
| <4.50 | 1.0 |  |  | 1.0 |  |  | 1.0 |  |
| ≥4.50 | 1.36 (1.21,1.54) | 0.001^**^ |  | 1.42 (1.25,1.62) | <0.001^***^ |  | 1.38 (1.22,1.57) | <0.001^***^ |
| BMI, body mass index; WC, waist circumference; WHtR, waist-to-height ratio; WHT.5R, waist divided by height^0.5^; BRI, body roundness index.  Model 1: adjusted by sex, age, Marital status.  Model 2: adjusted by sex, age, Marital status, smoking status, drinking status,Physical activity.  ^*^*P*-value < 0.05; ^**^*P*-value < 0.01; ^***^*P*-value < 0.001. | | | | | | | | |
