# Supplementary material for: Association of anthropometric indices with the development of multimorbidity in middle-aged and older adults: A retrospective cohort study
Source: PLoS One. 2022 Oct 14;17(10):e0276216. doi: 10.1371/journal.pone.0276216 (PMC9565419; doi:10.1371/journal.pone.0276216)
Supplement: S6 Table — (DOCX) [file pone.0276216.s007.docx]

| **S6 Table**  Association between WC and multimorbidity according to baseline characteristics. | | | | | | | |
| --- | --- | --- | --- | --- | --- | --- | --- |
| **Subgroup** | **Multimorbidity** | | **No-multimorbidity** | | **HR (95%CI)** | ***P*-value** | ***P*-value for interaction** |
|  | **WC** | | **WC** | |  |  |  |
|  | **<90 (male)/80 (female)** | **≥90 (male)/80 (female)** | **<90 (male)/80 (female)** | **≥90 (male)/80 (female)** |  |  |  |
| Age (years) |  |  |  |  |  |  | 0.826 |
| 45-59 | 541 | 109 | 16,560 | 1,418 | 1.87 (1.52,2.30) | <0.001^***^ |  |
| 60-74 | 3,397 | 733 | 36,754 | 4,937 | 1.45 (1.34,1.57) | <0.001^***^ |  |
| 75-85 | 921 | 185 | 8,331 | 1,142 | 1.37 (1.17,1.60) | <0.001^***^ |  |
| Sex (n (%)) |  |  |  |  |  |  | 0.082 |
| Male | 2,454 | 144 | 29,895 | 768 | 1.93 (1.63,2.28) | <0.001^***^ |  |
| Female | 2,405 | 883 | 31,750 | 6,729 | 1.53 (1.41,1.65) | <0.001^***^ |  |
| Marital status |  |  |  |  |  |  | 0.912 |
| Single | 513 | 120 | 7,220 | 926 | 1.56 (1.28,1.91) | <0.001^***^ |  |
| Couple | 4,346 | 907 | 54,425 | 6,571 | 1.53 (1.42,1.64) | <0.001^***^ |  |
| BMI (kg/m^2^) |  |  |  |  |  |  | 0.377 |
| <24 | 2,170 | 102 | 34,534 | 1,070 | 1.32 (1.08,1.61) | 0.006^**^ |  |
| 24.0-28.0 | 2,148 | 448 | 23,657 | 3,573 | 1.32 (1.19,1.46) | <0.001^***^ |  |
| ≥28.0 | 541 | 477 | 3,454 | 2,854 | 1.08 (0.96,1.22) | 0.208 |  |
| WHtR |  |  |  |  |  |  | 0.126 |
| <0.5 | 1,885 | 1 | 29,388 | 2 | 4.92 (0.69,34.90) | 0.111 |  |
| ≥0.5 | 2,974 | 1,026 | 32,257 | 7,495 | 1.39 (1.29,1.49) | <0.001^***^ |  |
| WHT.5R |  |  |  |  |  |  | 0.818 |
| <6.76 | 3,559 | 4 | 50,138 | 48 | 1.06 (0.40,2.83) | 0.904 |  |
| ≥6.76 | 1,300 | 1,023 | 11,507 | 7,449 | 1.19 (1.10,1.29) | <0.001^***^ |  |
| BRI |  |  |  |  |  |  | 0.714 |
| <4.84 | 3,446 | 50 | 48,187 | 506 | 1.26 (0.95,1.66) | 0.110 |  |
| ≥4.84 | 1,413 | 977 | 13,458 | 6,991 | 1.27 (1.17,1.38) | <0.001^***^ |  |
| Smoking (n (%)) |  |  |  |  |  |  | 0.374 |
| No | 4,585 | 999 | 58,934 | 7,298 | 1.54 (1.44,1.65) | <0.001^***^ |  |
| Yes | 274 | 28 | 2,711 | 199 | 1.31 (0.89,1.93) | 0.176 |  |
| Drinking (n (%)) |  |  |  |  |  |  | 0.211 |
| No | 4,550 | 981 | 58,769 | 7,265 | 1.53 (1.43,1.64) | <0.001^***^ |  |
| Yes | 309 | 46 | 2,876 | 232 | 1.66 (1.22,2.26) | 0.001^**^ |  |
| Physical activity (n(%)) |  |  |  |  |  |  | 0.150 |
| No | 1,896 | 346 | 30,690 | 3,181 | 1.48 (1.32,1.66) | <0.001^***^ |  |
| Yes | 2,963 | 681 | 30,955 | 4,316 | 1.53 (1.41,1.66) | <0.001^***^ |  |
| Abbreviations: BMI, body mass index; WC, waist circumference; WHtR, waist-to-height ratio; WHT.5R, waist divided by height^0.5^; BRI, body roundness index.  ^*^*P*-value < 0.05; ^**^*P*-value < 0.01; ^***^*P*-value < 0.001 | | | | | | | |
